# Parametric Optimisation of Friction-Stir-Spot-Welded Al 6061-T6 Incorporated with Silicon Carbide Using a Hybrid WASPAS–Taguchi Technique

**DOI:** 10.3390/ma15186427

**Published:** 2022-09-16

**Authors:** Neeru Chaudhary, Sarbjit Singh, Mohinder Pal Garg, Harish Kumar Garg, Shubham Sharma, Changhe Li, Elsayed Mohamed Tag Eldin, Samah El-Khatib

**Affiliations:** 1 Department of Mechanical Engineering, Punjab Engineering College (Deemed to be University), Chandigarh 160012, India; 2 Department of Mechanical Engineering, DAV University, Jalandhar 144001, India; 3 Mechanical Engineering Department, University Center for Research & Development, Chandigarh University, Mohali 140413, India; 4 School of Mechanical and Automotive Engineering, Qingdao University of Technology, Qingdao 266520, China; 5 Faculty of Engineering and Technology, Future University in Egypt, New Cairo 11835, Egypt

**Keywords:** friction stir spot welding, SiC microparticles, MCDM, WASPAS, Taguchi

## Abstract

Friction stir spot welding (FSSW) is one of the most popular fusion joining processes. The process is a solid-state welding process that allows welding of weldable as well as non-weldable materials. As a part of this investigation, weld samples of Al6061-T6 were reinforced with silicon carbide (SiC) powder with an average particle size of 45 µm. Initially, a Taguchi L9 orthogonal array was developed with three factors, i.e., rotational speed of the tool, pre-dwelling time, and diameter of the hole that was filled with SiC before welding. The effects of the SiC particles and process parameters were investigated as tensile–shear load and micro-hardness. The optimisation of parameters in order to maximise the output responses—i.e., strength and hardness of the welded joints—was performed using a hybrid WASPAS–Taguchi method. The optimised process parameters obtained were a 3.5 mm guiding hole diameter, 1700 rpm tool rotation speed, and 14 s of pre-dwelling time.

## 1. Introduction

**Figure 1:**
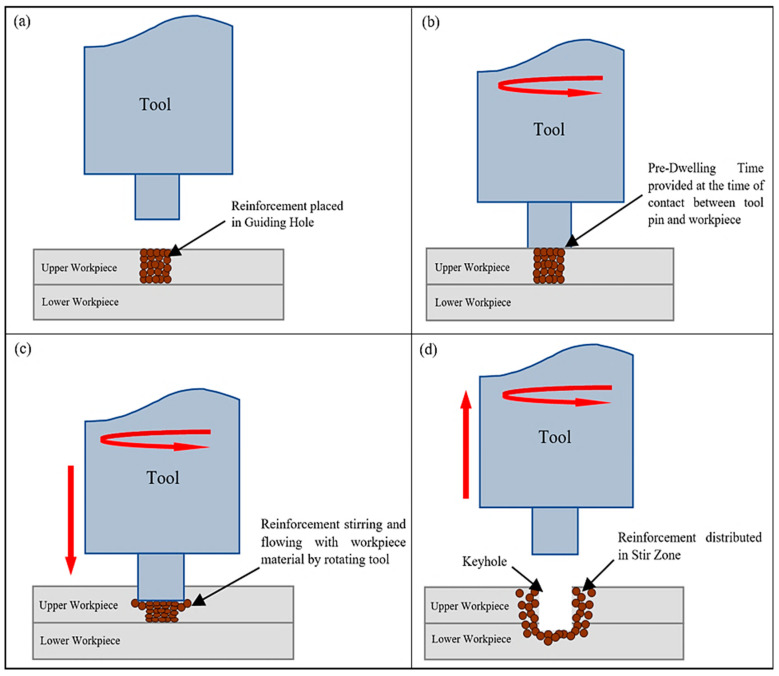
Schematic of the FSSW process: (**a**) placing reinforcement in a predrilled hole; (**b**) pre-dwelling time; (**c**) plunging and stirring; (**d**) retracting.

In industries such as the automotive and aerospace sectors, reduction in fuel utilisation and the emission of harmful gases is one of the priorities for years to come. This can be achieved by using lightweight materials, such as aluminium and its alloys, instead of traditional iron-based alloys. Amongst various joining techniques, initially, resistance spot welding (RSW), laser welding, etc., were employed for joining aluminium and its alloys [1,2]. However, these welding processes have many disadvantages, such as porosity, cracking, severe wear of the electrode tip, etc. As a result, various industries were on the lookout for alternative methods, and the automobile industry came up with a process known as friction stir spot welding (FSSW). FSSW is a most promising method for joining various materials, because it does not add weight to the material to be joined, and is also a cost-effective method [3,4]. FSSW starts with plunging the rotating tool into the workpieces, and after reaching a predefined depth the tool rotates and stirs the material in the stirring zone. During stirring, the temperature of the workpiece material rises due to friction, and reaches a value where the workpiece becomes soft and plastically deformed. When this process is ideally employed, the mixing of the workpiece material takes place without any significant change in its phase and microstructure, proving that the process is a solid-state joining process [5]. This can be attributed to the fact that the melting of the material leads to coarsening of the grains as grain boundaries break down during melting, and when the material cools down, those grains combine to form bigger grains, affecting the material properties. However, during the FSSW process, the material only deforms plastically, and finer grains are formed. A schematic of the FSSW process is shown in Figure 1. The quality of the weld produced by the FSSW process depends on various parameters, such as plunge depth, tool rotation speed, dwell time, plunge rate, tool configuration, etc.

Many research investigations have been carried out on the effects of process parameters on the output characteristics of welded joints, such as micro-hardness, tensile–shear load, etc. Uematsu et al. [6] described an inverse relationship of tool rotation speed and tool holding time with respect to tensile–shear strength, i.e., tensile–shear strength improved with the decrease in tool rotation speed and increase in tool holding time. However, cross-tension strength was inversely proportional to both. Gerlich et al. [7] also made a similar observation about tool rotation speed and strain rate, i.e., the weld strength increased with the decrease in tool rotation speed and increase in strain rate. In addition, they observed that size of the welded area did not change at higher tool rotation speeds. Conversely, Lathabai et al. [8] and Yuan et al. also [9] noted a bell-shaped curve between weld strength and tool rotation speed. Hence, higher tool rotation speed resulted in lower weld strength. This may be attributed to the increased heat input with the increase in the tool rotational speed, leading to coarsening of the grains in the weld zone, which resulted in reduced weld strength [10].

During FSSW, the tool experiences thrust forces while plunging into the workpiece, leading to wear of the tool and damage to the microstructure of the workpiece material. Hence, to reduce this damage and improve the performance of the process, researchers have investigated different methods of preheating workpieces before starting the plunging stage [11]. Shen et al. [12] used resistance heating rods for preheating, and observed an increase in the bonding area and reduction in the number of voids. Another investigation was carried out by Shen et al. [13] on the effects of preheating and different configurations of dissimilar aluminium–magnesium (Al–Mg) workpieces on weld strength. It was concluded that weld strength was improved with preheating in the case of Mg as the upper workpiece because of increased bond width, whereas in the case of Al as the upper workpiece, a decrease in weld strength was observed because of increased intermetallic compounds as compared to having Mg on top. Another method of preheating—i.e., heating with induction coil—was used by Sun et al. [14], and an enhancement in weld strength was observed. Hence, it can be stated that preheating assists in improving the mechanical properties of FSSW. However, the processes used for preheating the FSSW require special equipment, making the process cumbersome and costly. Therefore, this issue needs consideration, in order to provide a technique to preheat the weld in a simple and cost-effective manner.

It can be concluded from the literature that improving weld strength is priority, and in order to achieve this, the incorporation of reinforcements in the weld zone during FSSW has been found to be a reliable option. Initially, friction stir processing was used to alter the surface properties of materials such as brass, aluminium, etc., using nano- and micro-reinforcement particles. The use of these particles resulted in a substantial reduction in the grain size of the welded samples [15,16,17]. After that, the technique was used for improving the performance of FSSW. Researchers have reported different studies on the effects of distribution of reinforcements such as B_4_C, SiC, etc., combined with other parameters such as tool rotation speed, dwell time, etc., on the mechanical and microstructural properties of FSSW [18]. Among various reinforcements, SiC is considered a valuable contender because of its properties, such as lower thermal expansion coefficient, higher melting point, etc. Investigations have been carried on FSSW of aluminium alloys [19], magnesium alloys [20], copper [21], and other materials reinforced with silicon carbide during welding, and researchers have observed an increase in weld strength as well as the micro-hardness of welds, with homogeneous distribution of SiC particles in the welded area. Hence, it can be concluded that the incorporation of SiC particles in FSSW is a prominent method of enhancing the weld properties. However, the incorporation of SiC particles alone is not enough; their homogeneous distribution in the welded area is also a major contributor. This can be achieved by proper stirring and mixing of the SiC particles with plastically deformed workpiece materials by selecting appropriate process parameters.

With regards to the above discussion, it is clear that tool rotation speed, along with preheating or pre-dwelling time, helps in achieving material flow by generating the required amount of heat via friction, which can help in homogeneous mixing of reinforcements in the welded area, and serves the purpose of improving weld performance. However, selecting the right combination of levels of these parameters is a complicated task. Hence, optimisation of parameters is necessary to simplify the selection of parameters to obtain improved results. Therefore, various mathematical and statistical models have been developed in order to reduce the human resources and time consumed during experimentation [22]. Acharya et al. [23] welded dissimilar materials using FSSW and optimised the process parameters using the Taguchi method. Bozkurt and Bilici [24] also used the Taguchi method to optimise the process parameters to weld dissimilar aluminium alloys. An attempt was made by Bilici et al. [25] to optimize FSSW tool materials and process parameters using the Taguchi method. Meanwhile, Pradhan et al. [26] attempted a hybrid RSM–WASPAS–grey wolf technique to determine the optimal process parameters for dissimilar FSSW.

The Taguchi method is a robust statistical tool that is frequently used for optimising and analysing industrial processes. The Taguchi method can be applied for cost-effective system design, and it also helps to understand the impact of individual and combined process parameters [25]. Meanwhile, multi-criteria decision-making (MCDM) has proven to be a dynamic decision-making method that considers several factors in order to choose the appropriate process parameters. Hence, both techniques have proven to be better and more economical techniques for optimisation. However, there is limited literature available on the optimisation of the FSSW process using MCDM or hybrid MCDM–Taguchi techniques. 

This paper presents a combined technique of the Taguchi method and multi-criteria decision making (MCDM)—i.e., weighted aggregated sum product assessment (WASPAS)—to optimise process parameters and analyse the results obtained at optimal values. The best-suited hybrid MCDM–Taguchi model—i.e., the WASPAS–Taguchi model—was established for understanding the effects and importance of process parameters on output quality characteristics, i.e., tensile–shear load and micro-hardness.

## 2. Materials and Methods

### 2.1. Workpiece Material and Process Parameters

**Figure 2:**
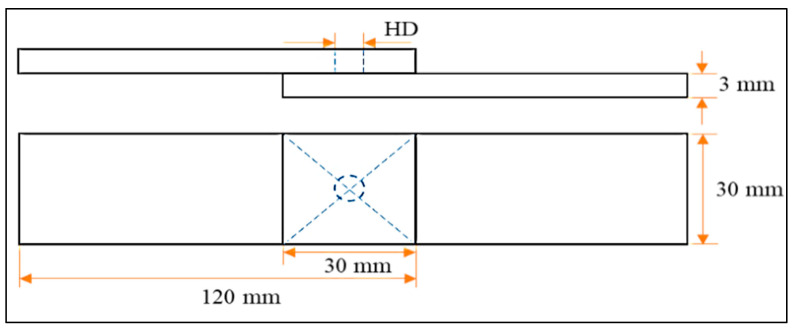
Test specimen configuration.

In the present study, commercial grade Al6061-T6 with 120 mm × 30 mm × 3 mm dimensions was chosen as the workpiece material, and measured test specimens were prepared using a diamond cutter. The weld specimens were retained in fixtures with an overlap of 30 × 30 mm^2^, as shown in Figure 2. Silicon carbide (SiC) particles with an average particle size of 45 µm were used for reinforcement. The reinforcement was positioned in predrilled guide holes before starting the welding process. Different diameters of the guiding holes—e.g., 2.5, 3.0, and 3.5 mm—were used to vary the quantity of SiC. In addition to the quantity of reinforcements, the tool rotational speed, pre-dwelling time, dwell time, etc., were taken as process parameters, the values of which are shown in Table 1. 

### 2.2. Tools and Methods

**Figure 3:**
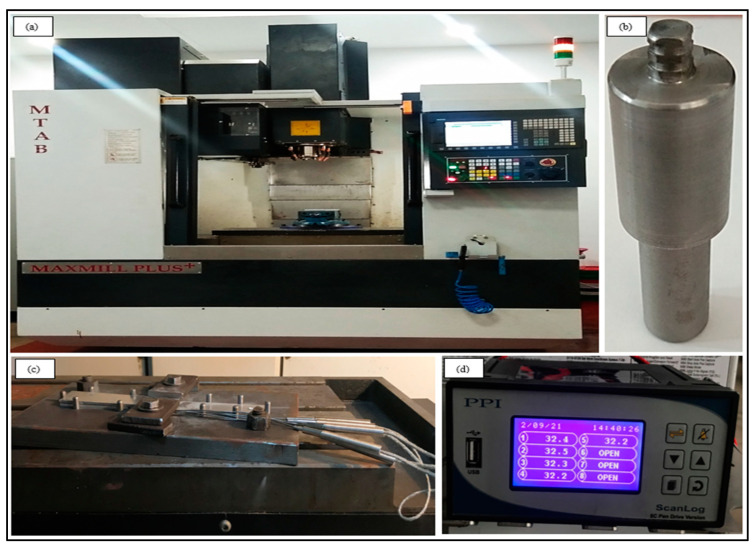
Experimental setup: (**a**) CNC milling machine; (**b**) FSSW tool; (**c**) fixture and thermocouple arrangement; (**d**) data logger.

**Figure 4:**
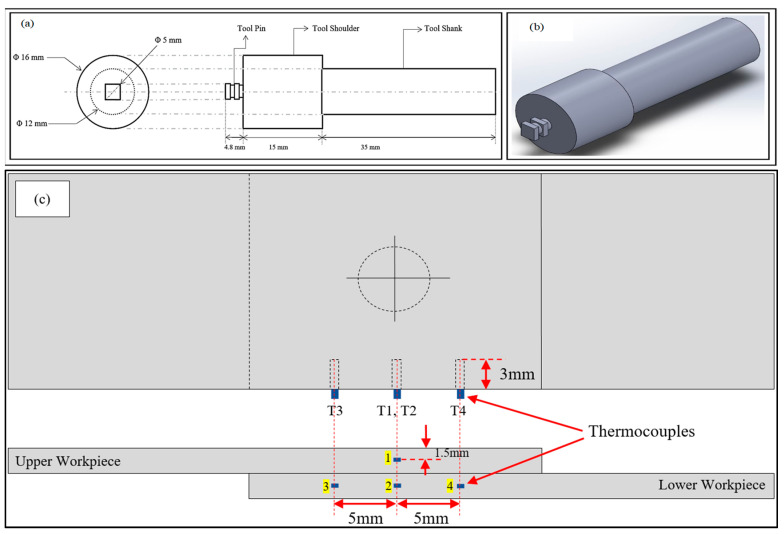
(**a**) Schematic of the FSSW tool; (**b**) 3D view of the FSSW tool; (**c**) thermocouple positions.

The experimentation was carried out at Siemens’ Center of Excellence in Manufacturing in a computer-numerical-controlled vertical machining centre, as shown in Figure 3. The FSSW tool used was made of high-carbon high-chromium steel with an average hardness of HRC 58, and with the shoulder having a concavity angle of zero. The tool pin was square-shaped, 4.8 mm long, and 5 mm in diameter (d), with grooves on it, while the tool shoulder was 16 mm in diameter (D), as shown in Figure 4a,b. Micro-hardness and tensile–shear load were calculated as response outputs. The welded samples were fractured on a universal testing machine (UTM, Fuel Instruments & Engineers Pvt. Ltd., Tal, India) (model UTE 40 HGFL with 40 KN load cell) at a speed of 1 mm/min by gradually increasing the load, and the corresponding tensile–shear load was measured. The experiments were repeated thrice in order to maintain the accuracy of the measured output quality characteristics. Additionally, the micro-hardness of the weld samples was measured using the Vickers micro-hardness measuring device (model HV 1000 B) at load of 100 g for a dwell time of 20 s. The macrostructural and microstructural behaviour of the welded joint was examined with distinct tools and techniques, such as scanning electron microscopy (SEM, model: JEOL JSM-IT500 LV with direct magnification of 5× to 300,000×), stereo-zoom microscopy (Stemi 508 with magnification capacity between 2× and 250×), energy-dispersive spectroscopy (EDS, model Oxford instruments Ultim Max with element detection range of Be (4)–Am (95)), and optical microscopy (OM, model Zeiss Axiocam ICc 1) etc. Sample preparation for the study was prepared by polishing samples with different emery papers and then polishing them with Brasso using a velvet cloth to obtain a scratchless surface. Temperature measurement was carried out using a K-type thermocouple and a data logger. Thermocouples were placed in four pre-drilled holes in the workpieces, highlighted in yellow in Figure 4c, and the obtained temperature readings were recorded using the universal data logger (Figure 3d). The optimisation model was developed using MS Excel.

### 2.3. Design of Experiments

The Taguchi method has proven to be a simple and efficient tool for designing experiments that have applications in various areas. This technique helps in reducing the cost of research by using an optimal orthogonal array, without affecting the efficiency of the process. Since the present research consisted of three input parameters with three levels, an L9 orthogonal array (OA) design was designated according to the Taguchi design of experiments. The experimental design according to the L9 orthogonal array is shown in Table 2.

## 3. Results and Discussions

### 3.1. Temperature Profile

The temperature was monitored at four different sites; two of them were placed in the centre line of the guiding hole in the upper workpiece (T1), while the other two were placed in the lower workpiece (T2) at a distance of 5 mm on either side of T2. The temperature was monitored as soon as the pre-dwelling stage began, and it was found that the temperature rose significantly during the pre-dwelling phase due to friction that occurred between the tool and the upper surface of the workpiece, as well as the SiC particles. After that, the tool plunged into the workpieces, and the temperature continued to rise as a result of friction between the rotating tool and the stirred material. After reaching the highest temperature (peak) possible during the stirring stage, the temperature began to fall gradually until it reached room temperature after retraction of the tool.

**Figure 5:**
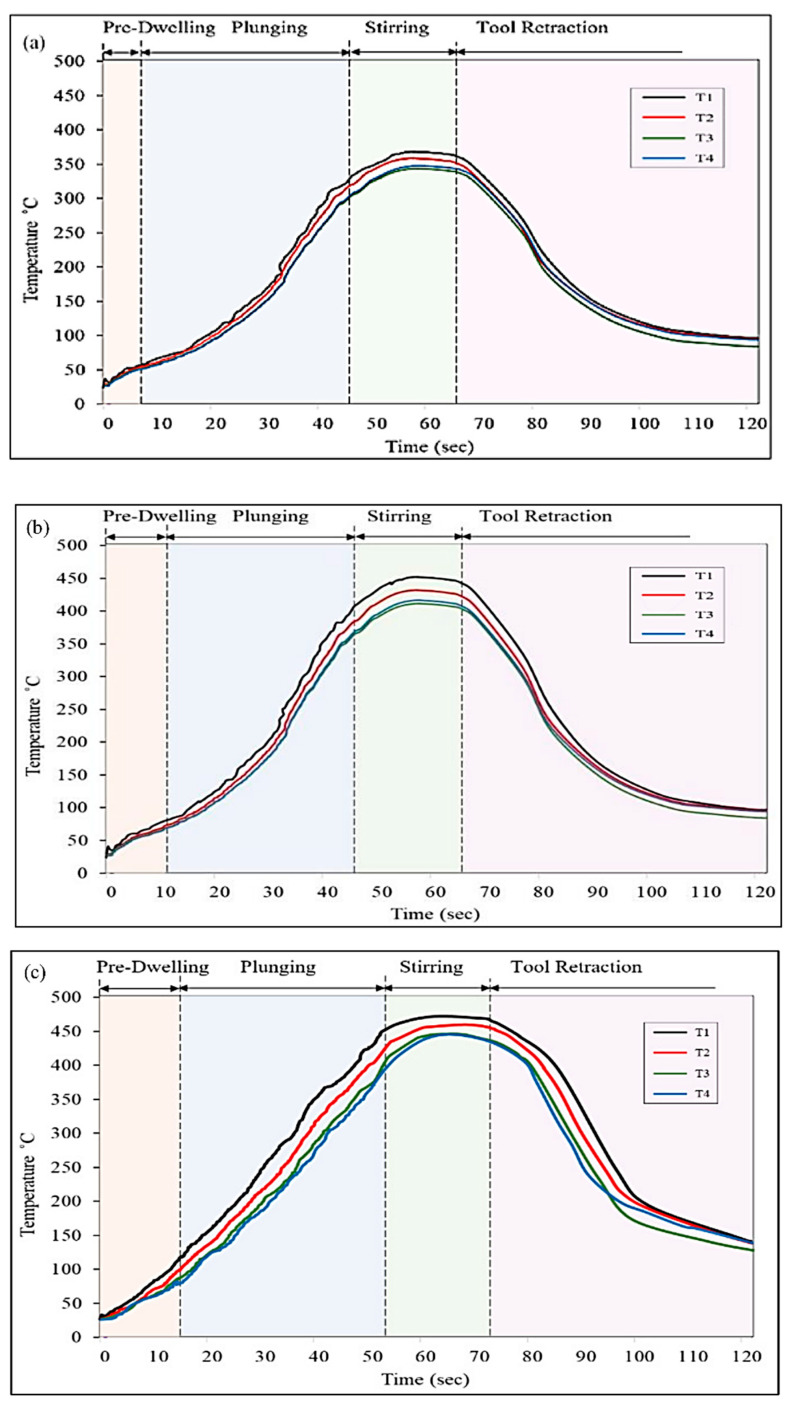
Temperature profile of the FSSW welds obtained at (**a**) 2.5 mm guiding hole diameter, 1300 rpm tool rotation speed, and 6 s of pre-dwelling time; (**b**) 3.5 mm guiding hole diameter, 1300 rpm tool rotation speed, and 14 s of pre-dwelling time; and (**c**) 3.5 mm guiding hole diameter, 2100 rpm tool rotation speed, and 10 s of pre-dwelling time.

Different temperature profiles were obtained with different combinations of process parameters. The temperature profiles obtained during FSSW of the Al6061-T6 welds are shown in Figure 5. It can be observed from the curves that the temperature obtained with a 2.5 mm guiding hole diameter, 1300 rpm tool rotation speed, and 6 s of pre-dwelling time was 379 °C, while a temperature of 442 °C was obtained for the weld produced with a 3.5 mm guiding hole diameter, 1300 rpm tool rotation speed, and 14 s of pre-dwelling time. However, the weld produced with a 3.5 mm guiding hole diameter, 2100 rpm tool rotation speed, and 10 s of pre-dwelling time reached the highest temperature, at 460 °C. The temperature curves of the different thermocouples indicate that the increase in temperature at the point near the upper surface of the upper workpiece was greater than that at the other three points. This may be explained by the fact that the material directly below the tool shoulder, also known as point T1, suffered plastic deformation over a wider region, which led to a greater temperature increase when compared to the other sites. When the tool first made contact with the workpiece, the temperature curve exhibited some fluctuation for a short period of time, but after that, the curve rose smoothly, and continued to do so until the tool started plunging. During plunging of the tool in the workpieces, the temperature profile showed fluctuations, and the observations were consistent with those of Ilman et al. [27]. The authors are of the opinion that the FSSW process is similar to the drilling process, which involves plunging of a rotating tool into the material of the workpiece and the generation of unequal and significant forces; hence, the fluctuation in the temperature profile was obtained until the stirring stage. However, after the tool reached the predetermined depth, the fluctuation reduced, and a smooth curve with peak temperature was attained, after which the temperature declined after the retraction of the tool.

**Figure 6:**
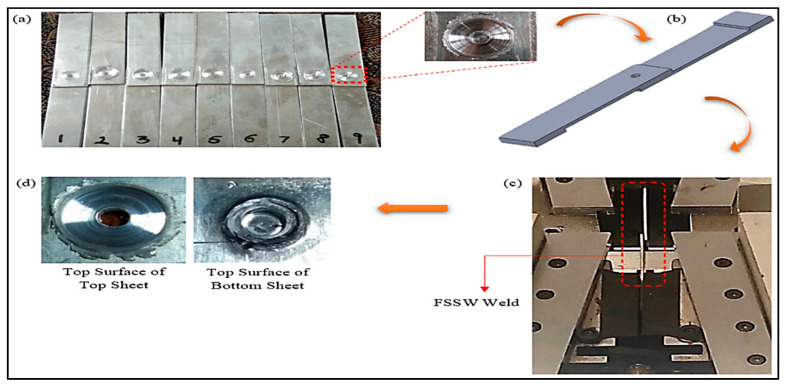
(**a**) 9 FSSW specimens; (**b**) FSSW test sample, (**c**) test sample secured in the UTM; (**d**) fractured sample.

**Figure 7:**
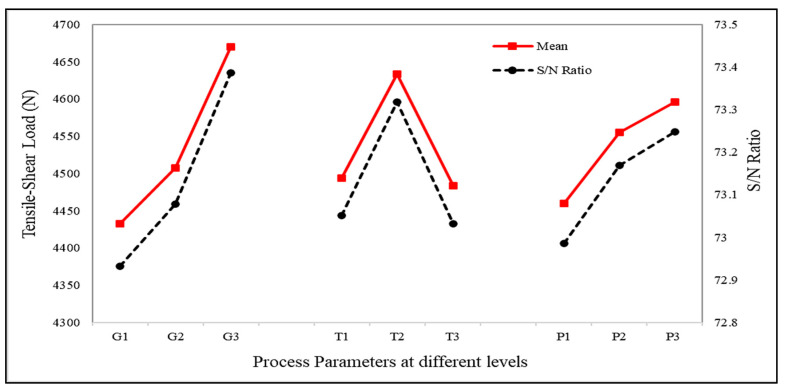
Effects of the process parameters on tensile–shear load.

**Figure 8:**
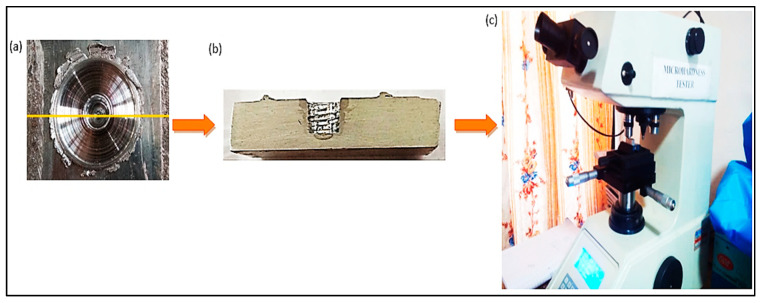
(**a**) FSSW weld; (**b**) FSSW weld cut in half; (**c**) micro-hardness tester.

**Figure 9:**
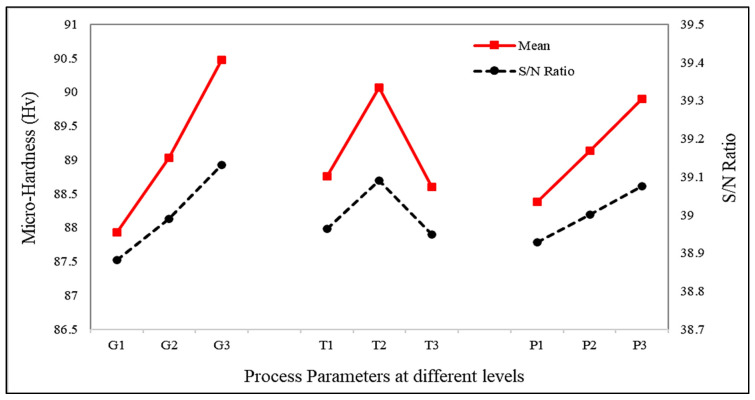
Effects of process parameters on micro-hardness.

**Figure 10:**
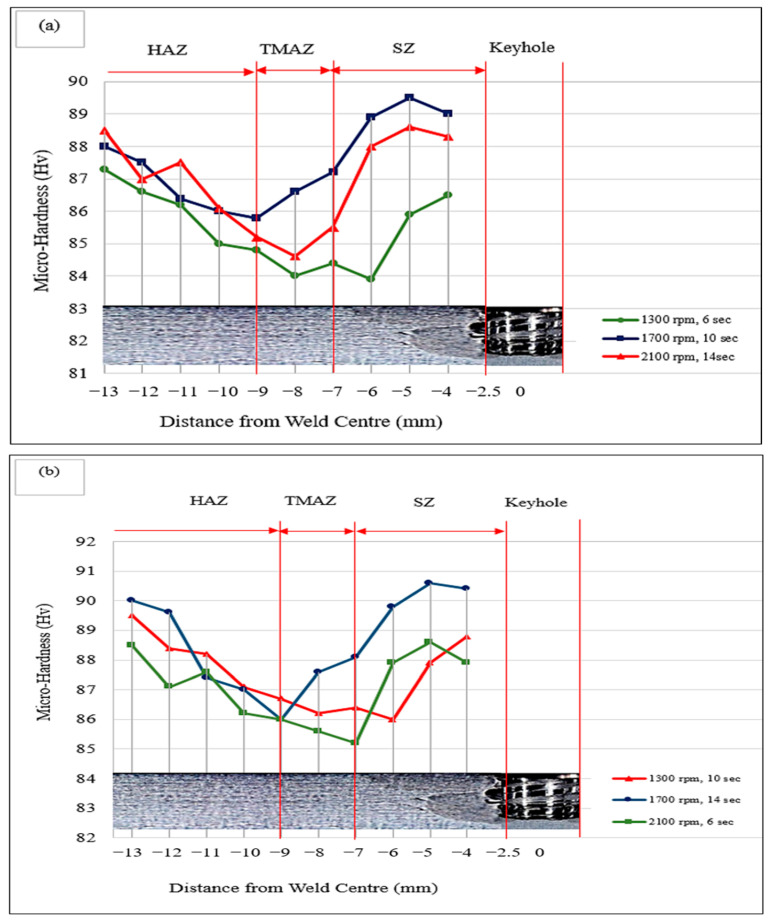

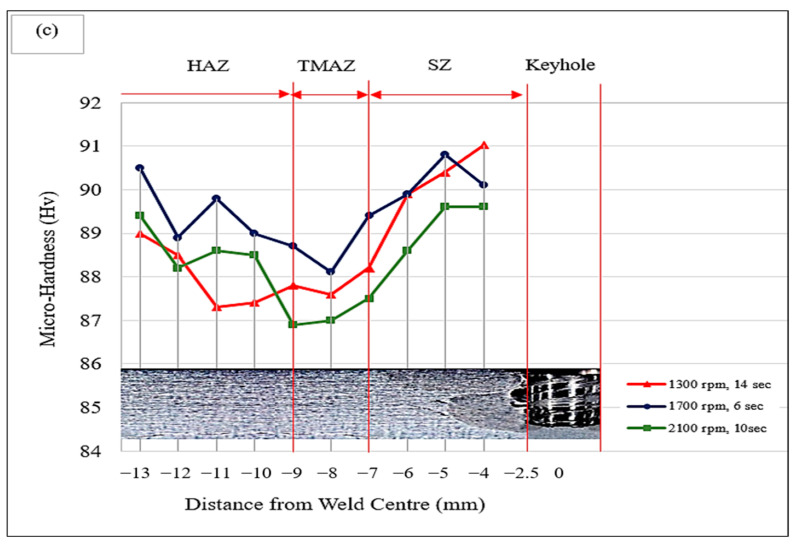
Micro-hardness profiles of samples obtained at (**a**) 2.5 mm guiding hole diameter, (**b**) 3.0 mm guiding hole diameter, and (**c**) 3.5 mm guiding hole diameter.

The effect of pre-dwelling time on temperature can clearly be seen from the temperature profiles. As the pre-dwelling time increased from 6 s (Figure 5a) to 10 s (Figure 5b), and then to 14 s (Figure 5c), the temperature attained by the end of this stage also increased due to prolonged friction. This rise in temperature increased the heat input and made it easier to plunge the tool into the workpieces; hence, comparatively less fluctuation can be seen in Figure 5b,c. Hence, pre-dwelling time assisted in increasing the overall temperature during the process and obtaining the required heat and material flow. Another finding in terms of SiC quantity is that the temperature increased with the increase in the guiding hole’s diameter, i.e., by increasing the quantity of SiC, which may be attributed to the fact that increasing quantity of SiC enhanced the frictional heating due to the hard nature of the SiC particles. Another parameter affecting the temperature profile was tool rotation speed. Frictional heating increased with the increase in the tool’s rotation speed; hence, the temperature of the workpiece also increased with the increase in the tool’s rotation speed. It can be concluded from Figure 5 that the temperature of the weld (Figure 5a) at a guiding hole diameter of 2.5 mm was less than that of the other two welds with a guiding hole diameter of 3.5 mm. The peak temperatures shown in Figure 5b,c do not differ significantly. Hence, it can be concluded that the guiding hole diameter significantly affected the increase in temperature in the welds.

### 3.2. Behavioural Analysis of FSSW Welds in Terms of Tensile–Shear Load

A pictographic demonstration of the steps involved during tensile–shear analysis of weld samples is shown in Figure 6. The welded samples were made equiaxed by gluing two parts of equivalent dimensions at the edges of both workpieces in order to hold the welded samples in the UTM, aligned in a straight line. Then, a progressively increasing load was applied, and the average of three experimental values of tensile–shear load for each experiment of the Taguchi L9 orthogonal array was recorded, as shown in Table 2. 

A graphical representation of how the tensile–shear load varied with variation in the process parameters is shown in Figure 7. It was observed that the tensile–shear load initially increased and then decreased with increasing tool rotation speed. In order to evaluate the effects of different parameters on the tensile–shear load, the mean tensile–shear load of each parameter in the different experiments was calculated and correspondingly plotted in graphs, as shown in Figure 7. The tensile–shear load first increased to 4633.56 N at 1700 rpm, and then decreased to 4482.17 N at 2100 rpm. The increase in the tool rotation speed resulted in increased heat input due to friction. This increased heat input softened the workpieces, facilitating the flow of material and the mixing of the two workpiece materials. Throughout the process, grain refinement took place due to stirring of the material in the stir zone (SZ). Consequently, a finer microstructure was achieved in the SZ, which increased the elongation of joints during tensile–shear testing; hence, the joints fractured at a higher load. During this process, the workpieces were heated up, and took longer to cool down, as shown in Figure 5. The temperature increase at 2100 rpm was greater than at the other two tool rotation speeds. Hence, the greater temperature increases and consequent delay in the cooling of the material led to growth of the grain boundaries and consequent coarsening of grains. This reduced the ductility of the joints, leading to early fracture of the weld. This behaviour of the rotation speed is comparable to that found in previous investigations [9].

On the other hand, weld strength considerably improved with an increase in the guiding hole diameter, i.e., with increase in the amount of SiC particles. An increase in tensile–shear load from 4432.23 N to 4670.4 N with the increase in the guiding hole’s diameter from 2.5 mm to 3.5 mm was recorded during our investigation. When the tool penetrated the workpieces, the reinforcements were mixed with the material in the stir zone. The presence of SiC particles increased the frictional heat input due to their high hardness. However, the increase in the heat input did not lead to grain growth in the base material, because of the lower thermal coefficient of the SiC particles. This property of the SiC particles prevented their expansion due to the increase in heat input, and also acted as a barrier to other grains present in the vicinity. Therefore, when the quantity of SiC was increased, the resistance to grain growth also increased. Hence, a finer microstructure was obtained in the stir zone, making the FSSW joints stronger. Secondly, grain refinement occurred due to the higher number of nucleation sites. SiC particles act as nucleation sites; hence, the higher the number of SiC particles, the higher the number of nucleation sites and the greater the opposition to grain growth. All of these factors contributed to enhancing the weld strength and confirmed the positive influence of SiC on FSSW quality. This behaviour of weld strength with respect to SiC is similar to the findings of previous research [17].

Similarly, a direct proportional relationship was witnessed between weld strength and pre-dwelling time. Weld strength increased from 4459.34 N to 4596.02 N with the increase in pre-dwelling time from 6 s to 14 s. This was due to the fact that contact time between the tool pin and the upper workpiece increased with pre-dwelling time, and increased the heat input due to prolonged friction. This process softened the upper workpiece and made it easier to plunge the tool into the workpieces and develop sufficient material flow. The induced material flow around the tool pin led to mixing of the workpieces, and resulted in improved tensile–shear load. These findings have been verified and justified by previous research related to FSSW of aluminium alloys [12].

### 3.3. Behavioural Analysis of FSSW Welds in Terms of Micro-Hardness

A pictographic demonstration of the micro-hardness testing of the welded specimens is shown in Figure 8. The FSSW weld was first cut from middle, and then a cross-section was finished using emery paper. After achieving a good surface finish, the sample was placed on a micro-hardness tester, and values of micro-hardness at the distance of 4 mm from the centre of the keyhole—up to 13 mm on one side and 2.0 mm below the top surface—were calculated. The average experimental values of micro-hardness in the three experiments for each Taguchi L9 orthogonal array are listed in Table 2, and the impact of the process parameters on micro-hardness is presented in Figure 9. It was observed that micro-hardness first increased with the tool rotation speed, and then reduced. While a directly proportional relationship was observed between micro-hardness and both guiding hole diameter and pre-dwelling time. This can be attributed to the Hall–Petch effect, where the grain size is inversely proportional to the hardness. According to the Hall–Petch equation [28], HV = H_0_ + k_H_d^−1/2^, where HV is hardness, d indicates grain size, and H_0_ and k_H_ are the constants; therefore, it can be seen from the equation that hardness decreases with the increase in grain size.

As discussed above, the increase in the tool rotation speed and the pre-dwelling time led to refinement of the grains in the stir zone (SZ). Therefore, enhanced micro-hardness was observed with the increase in these parameters. However, the value of micro-hardness first increased with tool rotation speed and then reduced, because of coarsening of the grains, which occurred due to increased heat input and a slower cooling rate. Moreover, the increase in hardness of the FSSW welds was mostly affected by presence of reinforcements in the stir zone, because the micro-hardness of a reinforced weld is correlated with the size of the grains, the presence of reinforcements, the density of dislocations, and the heat input [28]. The reinforcement particles in the stir zone acted as a hindrance for dislocations, and these dislocations were accumulated against the reinforcements. Hence, the dislocation density increased with the increase in the number of reinforcements in the stir zone, along with grain refinement that occurred due to pinning of the grain boundaries by SiC particles, which resulted in increased hardness. The change in hardness between different regions of the welds at with different process parameters is presented in Figure 10. It can be observed from the hardness profiles that the stir zone (SZ) had a higher hardness, which gradually decreased through the thermo-mechanically affected zone (TMAZ) and attained the minimum value. Thereafter, the hardness increased further toward the base metal, exhibiting a W-shaped appearance. All hardness profiles showed a higher Vickers micro-hardness near the keyhole, representing the SZ of dynamically recrystallised fine grains. The results given in Figure 10 show that the micro-hardness value was not uniform throughout the SZ. Hence, as per the Hall–Petch equation, tensile–shear strength and increased micro-hardness, the grain size reduced [28]. Fortuitously, the results of the tensile–shear tests obtained in this work are in excellent accordance with the micro-hardness profiles. The tensile–shear load and micro-hardness exhibited similar variations in relation to the process parameters.

## 4. Optimisation of Process Parameters Using the Hybrid WASPAS–Taguchi Technique

**Figure 11:**
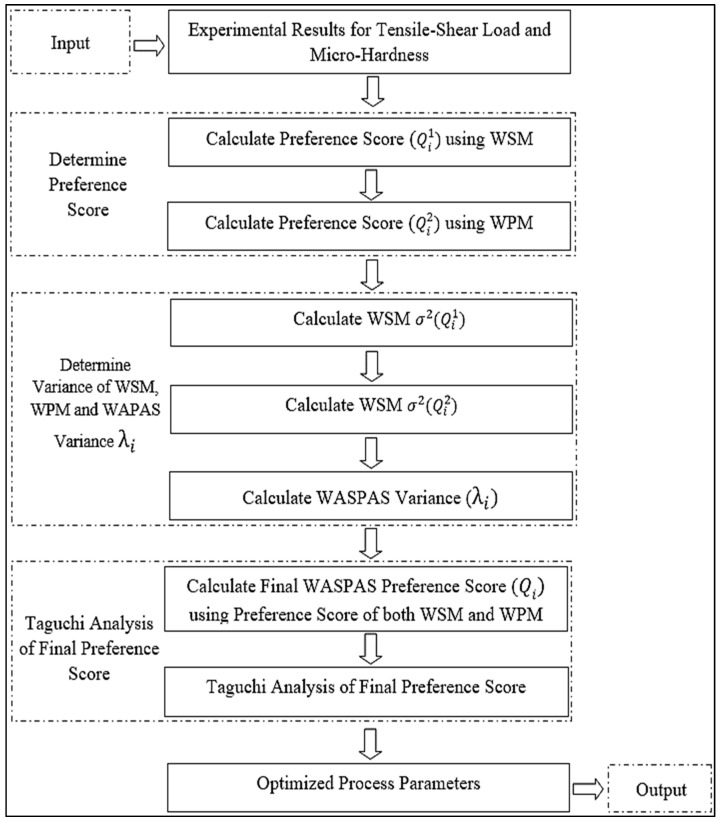
Flowchart of the hybrid WASPAS–Taguchi technique.

Multi-criteria decision-making (MCDM) methods have been used to address issues that require decision-making where there is more than one criterion. There are different methods that have been used to treat decision-making issues, and one of them is WASPAS. WASPAS is a multi-objective optimisation-making (MODM) method, which is one category of MCDM methods, and is used to determine the weight of output characteristics or alternatives, and to rank process parameters to choose the best alternatives among several. This is a combination of two well-established models—i.e., the weighted sum model (WSM) and the weighted product model (WPM)—that makes the proposed solution more stable. This technique works effectively and is compatible with other methods. WASPAS allows determination of weights according to the importance of each attribute, which helps in rational decision-making processes [29]. The present paper presents the optimisation of process parameters using WASPAS—an MCDM technique—combined with a very popular statistical analysis technique, known as the Taguchi method. Figure 11 shows the steps involved in the hybrid WASPAS–Taguchi technique.

### 4.1. Step 1: Determination of the Normalised Decision Matrix

The following steps were used to eliminate every irregularity in the experimental results by changing them to dimensionless quantities: The equation below was used for normalisation. Hence, the outcomes were obtained in a 0–1 range.

(1) $$\bar{x_{ij}}=\frac{x_{ij}}{\max x_{ij}}\;\mathrm{to}\;\mathrm{maximise}.$$

where *i* = 1, 2, 3 ……..., n and *j* = 1, 2, 3 …..., m; n = the number of criteria or responses (tensile–shear load and micro-hardness); m = the number of alternatives or the experiment number, to be ranked (Experiments 1–9). Table 3 shows the normalised decision matrix.

### 4.2. Step 2: Creation of the Performance Matrix by WSM and WPM

WASPAS has been described as the hybrid of two MCDM techniques, i.e., the weighted sum method (WSM) and the weighted product method (WPM). Hence, the total performance matrix is a combination of performance matrices for WSM and WPM. The performance matrix for WSM is given as follows [30]:

(2) $$\mathrm{WSM}=Q_{i}^{1}=\sum_{j-1}^{n}\bar{x_{ij}}*w_{j}$$

The performance matrix for WPM is given as follows:

(3) $$\mathrm{WPM}=Q_{i}^{2}=\prod_{j=1}^{n}{\bar{x_{ij}}}^{w_{j}}$$

where *w_j_* is the weight of the *j*^th^ alternative (Experiments 1–9). The performance matrix for WSM and WPM is shown in Table 4.

### 4.3. Step 3: Calculation of Variance 

Calculation of variance (σ^2^) is required for the final performance matrix, as errors occur during determination when the initial values of the criteria are stochastic or have a random probability distribution. Hence, calculation of σ^2^ is needed to determine the dispersal of outcomes in the distribution. Variance for the WSM and WPM can be calculated using Equations (4) and (5), respectively, and is shown in Table 5 [31,32,33].

For WSM:

(4) $$\sigma^{2}\left(Q_{i}^{1}\right)=\overset{n}{\underset{j=1}{\sum }}\sigma^{2}(\bar{x_{ij}})*w_{j}^{2}$$

For WPM:

(5) $$\sigma^{2}\left(Q_{i}^{2}\right)=\overset{n}{\underset{j=1}{\sum }}\sigma^{2}(\bar{x_{ij}})*{\left[\frac{\prod_{j=1}^{n}x_{ij}{}^{w_{j}}.*\left(w_{j}\right)}{x_{ij}{}^{w_{j}}*x_{ij}{}^{\left(1-w_{j}\right)}}\right]}^{2}$$

where $\;\sigma^{2}\left(\bar{x_{ij}}\right)$ is the variance of the normalised decision matrix at a 95% confidence interval, calculated using Equation (6):

(6) $$\;\sigma^{2}\left(\bar{x_{ij}}\right)=\left(0.05*\;\bar{x_{ij}}\right){\;}^{2}$$

### 4.4. Step 4: Determination of $\lambda_{i}$

Likewise, variance $\lambda_{i}$ also affects the dispersal of outcomes in the distribution, and it can be determined using Equation (7) [32,34,35,36]:

(7) $$\lambda_{i}=\frac{\sigma^{2}\left(Q_{i}^{2}\right)}{\sigma^{2}\left(Q_{i}^{1}\right)+\sigma^{2}\left(Q_{i}^{2}\right)}$$

### 4.5. Step 5: Creating the Final Performance Matrix

The final preference score can be determined using Equation (8), which combines the preference matrices of WSM and WPM, as shown in Table 6.

(8) $$Q_{i}=\lambda Q_{i}^{1}+\left(1-\lambda \right)Q_{i}^{2}$$

where λ = 0, 0.1,…,1; here, λ = 0 implies that the WASPAS method is converted to the WPM method, while λ = 1 converts the WASPAS method to the WSM method. 

### 4.6. Step 6: Taguchi Analysis of the Final Performance Matrix

Based on the results of the final performance matrix, it was determined that experiment number 7, obtained under the process parameters G_3_T_1_P_3_, with the maximum final performance score, delivered better results in terms of both output responses in comparison with other experiments. However, the final optimised values were derived using the Taguchi technique. For analysis using the Taguchi method, the final performance score from Table 6 was used to determine the S/N responses corresponding to the process parameters and their levels. The process parameter level with the largest S/N ratio, as shown in Table 7, was considered the optimal process parameter condition. Therefore, the process parameters G_3_T_2_P_3_—i.e., guiding hole diameter of 3.5 mm, tool rotation speed of 1700 rpm, and pre-dwelling time of 14 s—were considered the optimal process parameters. The difference between the maximum and minimum S/N ratios of the performance scores was 0.277 for guiding hole diameter, 0.149 for tool rotation speed, and 0.186 for pre-dwelling time, as shown in Table 7. These values provide information about factors that highly affect output characteristics. Therefore, the parameter with the highest numeric value—i.e., guiding hole diameter—had the greatest effect on the output responses, followed by pre-dwelling time and tool rotation speed. 

Similarly, analysis of variance (ANOVA) was conducted on the final performance scores for assessment of significant and non-significant process parameters, and also to determine the statistical significance of the process parameters with respect to the weld quality, as shown in Table 8. The level of significance considered for ANOVA was 5%, i.e., 95% confidence level. Moreover, ANOVA provided a clear vision of how the process parameters influenced the response, along with their level of significance [22,33,34]. Meanwhile, the relative power of each factor was indicated by the percentage contribution of each parameter, which is a function of sum of squares, in order to reduce the disparity in the experimental results. Percentage contribution also indicates variation in parameters, which can be reduced by precisely controlling the levels of the parameters. Hence, ANOVA showed that the guiding hole diameter had the greatest effect on the response characteristics, followed by tool rotation speed and pre-dwelling time, with percentage contributions of 52.85%, 19.52%, and 23.45%, respectively. Furthermore, the *p*-value of each parameter was less than 0.05, showing that the parameters had a significant effect on the responses. Hence, a 3.5 mm guiding hole diameter, 1700 rpm tool rotation speed, and 14 s of pre-dwelling time was found to be the optimal combination of process parameters using the specially established hybrid WASPAS–Taguchi model.

## 5. Confirmation Test and Comparison of FSSW with and without SiC Particles Obtained under Optimal Process Parameters

In order to verify the predicted optimal parameters (3.5 mm guiding hole diameter, 1700 rpm tool rotation speed, and 14 s of pre-dwelling time), experimental runs were carried out under optimal conditions, and both tensile–shear load and micro-hardness were calculated as output responses. The obtained values of tensile–shear load and micro-hardness were 5145 N and 101 Hv0.1, respectively, which were greater than the tensile–shear load and micro-hardness value of the other nine experiments. Hence, the authenticity of the optimised results obtained via the hybrid WASPAS–Taguchi method was verified. 

**Figure 12:**
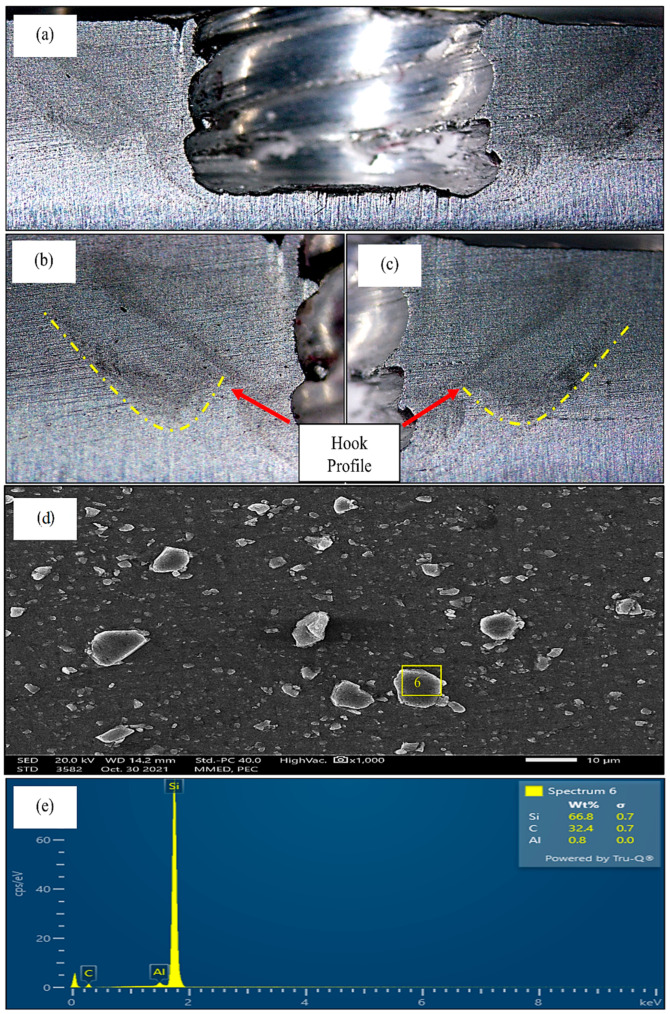
(**a**) Macrograph of the weld with SiC; (**b**) left side of the weld from the centre, with hook profile; (**c**) right side of the weld from the centre, with hook profile; (**d**) SEM image of the sample obtained with SiC; (**e**) EDS analysis.

The morphological analysis of FSSW under optimal parameters was carried out, and the results are shown in Figure 12. The macrograph of the weld showed a clear SZ and hook on either side of the keyhole, bent upwards like a mountain. A homogeneous distribution of SiC particles could be seen from the SEM analysis of the weld, as shown in Figure 12d. The SiC particles showed complete bonding with the base material, and EDS analysis showed evidence of their presence and the results are identical with the same [37,38,39]. This shows that the optimal parameters helped in inducing sufficient heat for the flow of the material and thorough mixing of the reinforcements and the matrix. There were no voids or partially bonded regions obtained. Hence, a stronger weld was obtained under optimal parameters as compared to the other nine experiments, confirming the success of the established model and these results are completely identical with the previous studies [40,41,42].

**Figure 13:**
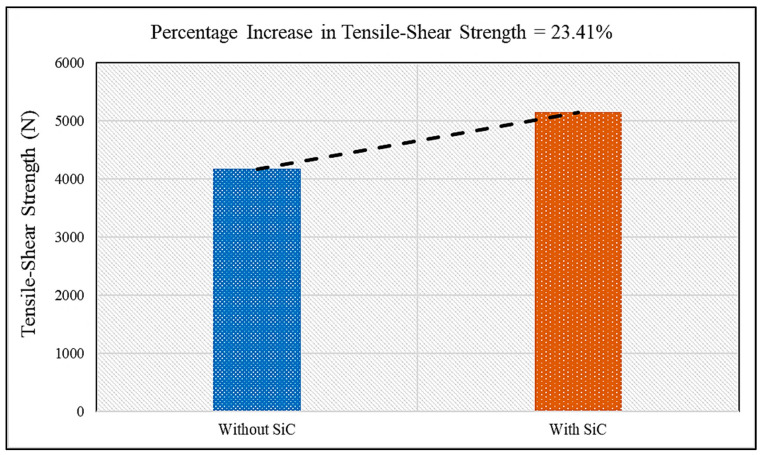
Comparative analysis of the tensile–shear load of FSS Welds without SiC and with SiC.

**Figure 14:**
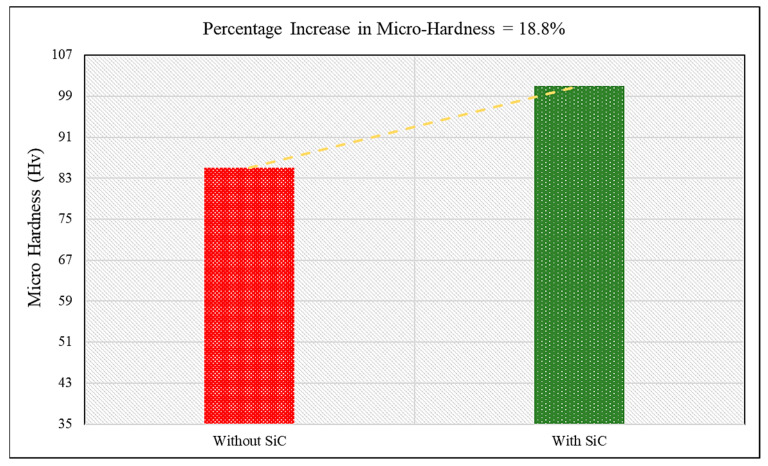
Comparative analysis of the micro-hardness of FSS welds without SiC and with SiC.

Thus, the objective of the present study was to analyse the consequences of adding SiC particles in conventional FSSW in terms of the mechanical and microstructural behaviour of the weld. To carry out our investigation, the results of FSSW with SiC were compared with conventional FSSW, both obtained under optimal conditions. The sample without SiC gave a tensile-shear load of 4169 N and micro-hardness of 85 Hv0.1; when compared with the results of welding with SiC, it was evident that the incorporation of SiC particles as reinforcement increased the weld strength by 23.41% and hardness by 18.8%, as shown in Figure 13 and Figure 14, respectively. The enhancement of the properties of the weld with SiC was obtained because of the amazing properties of reinforcement. The low thermal expansion coefficient of SiC offered resistance to the growth of aluminium grains when surrounded by SiC particles. In addition, the difference in the thermal expansion coefficients of SiC and aluminium led to the formation of strain fields or residual stress fields around the SiC particles during cooling of the weld which is comparable with the existing works [42,43,44]. The formation of strain fields led to piling up of dislocations, and when the welded samples were exposed to tensile–shear loading, the SiC particles and piled-up dislocations acted as a barricade and prevented crack propagation. Consequently, greater load was required to break through the SiC particles and accumulated dislocations. The increase in the number of dislocations occurred due to partial relief of the stresses caused by the different thermal expansion coefficients of SiC and the aluminium alloy [35,44,45]. Moreover, the SiC particles acted as a guard, and prevented damage to the aluminium alloy grains which shows similar findings with the existing works [46,47,48]. This process continued until the applied load increased to a level where the SiC–aluminium interface was damaged. It can be inferred that the fracture of the joint first started with damage to the SiC–aluminium interface, and then the crack propagated through the rest of the base material [36,49,50]. Furthermore, the increase in micro-hardness was due to the reduced grain size, as SiC particles help in obtaining a fine grain structure and, according to the Hall–Petch effect, hardness is inversely proportional to grain size; therefore, the micro-hardness of the weld with SiC particles was greater than that of the conventional weld. Additionally, the hardness of SiC particles is greater than that of grains of aluminium, which increased the combined hardness of the composite made in the SZ during welding.

## 6. Conclusions

In the present study, SiC-reinforced aluminium 6061-T6 FSSW welds were obtained. The effects of process parameters such as guiding hole diameter, tool rotation speed, and pre-dwelling time on output characteristics such as tensile–shear load and micro-hardness were observed. The following conclusions were obtained from the present research:

1. The guiding hole’s diameter plays a significant role in predicting the tensile–shear behaviour and micro-hardness of the joint.
2. The percentage contribution as analysed by the WASPAS–Taguchi method for guiding hole diameter was 52%, followed by pre-dwelling time (23%) and tool rotation speed (19%).
3. Based on the hybrid WASPAS–Taguchi method, G_3_T_2_P_3_—i.e., 3.5 mm guiding hole diameter, 1700 rpm tool rotation speed, and 14 s of pre-dwelling time—were the optimal values for both tensile–shear load and micro-hardness.
4. The FSSW with SiC particles exhibited increased tensile–shear load and micro-hardness under optimised process parameters.
5. An increase of 23.41% was observed in tensile–shear load, while micro-hardness increased by 18.8%, with the incorporation of SiC particles in FSSW, when compared with conventional FSSW, under optimal parameters.
6. Uniform distribution of SiC particles was observed in the SEM images.
7. The increase in SiC quantity significantly increased the peak temperature of the weld, due to the ceramic behaviour and lower conductivity of the SiC particles, which prevented the generated heat from escaping the welded region.

## Figures and Tables

**Table 1:** Process parameters and their levels.

Sr. No.	Process Parameters	Level 1	Level 2	Level 3
1.	Guiding Hole Diameter (mm)	2.5 (G_1_)	3.0 (G_2_)	3.5 (G_3_)
2.	Tool Rotation Speed (rpm)	1300 (T_1_)	1700 (T_2_)	2100 (T_3_)
3.	Pre-Dwelling Time (s)	6 (P_1_)	10 (P_2_)	14 (P_3_)
4.	Dwell Time (s)	20	20	20
5.	Plunge Rate (mm/min)	15	15	15
6.	Plunge Depth (mm)	4.8	4.8	4.8

**Table 2:** Process parameters and experimental results.

Expt. No.	Guiding Hole Diameter (mm)	Tool Rotation Speed (rpm)	Preheating Time (s)	Tensile–Shear Load (N) ± ST Dev.	Micro-Hardness (Hv 0.1) ± ST Dev.
1.	2.5	1300	6	4297.67 ± 13.78	86.50 ± 2.01
2.	2.5	1700	10	4562.79 ± 7.38	89.00 ± 1.25
3.	2.5	2100	14	4436.22 ± 10.85	88.70 ± 2.01
4.	3	1300	10	4480.55 ± 5.04	88.80 ± 1.47
5.	3	1700	14	4649.79 ± 3.04	90.30 ± 1.06
6.	3	2100	6	4392.25 ± 9.31	87.90 ± 1.43
7.	3.5	1300	14	4702.05 ± 7.44	91.02 ± 0.68
8.	3.5	1700	6	4688.10 ± 7.09	90.80 ± 1.86
9.	3.5	2100	10	4621.06 ± 9.07	89.60 ± 1.90

**Table 3:** Normalised decision matrix.

Expt. No.	Guiding Hole Diameter (mm)	Tool Rotation Speed (rpm)	Preheating Time (s)	Normalised Value	
				Tensile–Shear Load (N)	Micro-Hardness (Hv 0.1)
1.	2.5	1300	6	0.457	0.475
2.	2.5	1700	10	0.485	0.488
3.	2.5	2100	14	0.472	0.487
4.	3.0	1300	10	0.476	0.487
5.	3.0	1700	14	0.494	0.496
6.	3.0	2100	6	0.467	0.489
7.	3.5	1300	14	0.5	0.5
8.	3.5	1700	6	0.499	0.498
9.	3.5	2100	10	0.491	0.492

**Table 4:** Performance matrix for WSM and WPM.

Expt. No.	$\mathrm{Performance}\;\mathrm{Score}\;(Q_{i}^{1})$	$\mathrm{Performance}\;\mathrm{Score}\;(Q_{i}^{2})$
1.	0.932	0.217
2.	0.974	0.237
3.	0.958	0.229
4.	0.964	0.232
5.	0.990	0.245
6.	0.949	0.225
7.	1.000	0.250
8.	0.997	0.248
9.	0.983	0.242

**Table 5:** Variance for WSM and WPM.

Expt. No.	$\mathrm{Variance}\;(\sigma^{2}\left(Q_{i}^{1}\right))$	$\mathrm{Variance}\;(\sigma^{2}\left(Q_{i}^{2}\right))$
1.	0.001086	0.001174
2.	0.001186	0.001204
3.	0.001149	0.001226
4.	0.001162	0.001218
5.	0.001226	0.001234
6.	0.001128	0.001205
7.	0.001250	0.001250
8.	0.001243	0.001244
9.	0.001209	0.001213

**Table 6:** λ_i_, Final performance matrix and rank.

Expt. No.	Variance (λ_i_)	$\mathrm{Final}\;\mathrm{Performance}\;\mathrm{Score}\;(Q_{i})$	**Rank**
1.	0.5194	0.5885	9
2.	0.5038	0.6084	5
3.	0.5161	0.6062	7
4.	0.5117	0.6069	6
5.	0.5016	0.6190	3
6.	0.5166	0.5997	8
7.	0.5000	0.6250	1
8.	0.5002	0.6231	2
9.	0.5008	0.6133	4

**Table 7:** Response table of S/N ratios (the larger the better).

Level	Guiding Hole Diameter	Tool Rotation Speed	Preheating Time
1	−4.422	−4.341	−4.384
2	−4.341	**−4.196**	−4.299
3	**−4.145**	−4.345	**−4.198**
Delta	0.277	0.149	0.186
Rank	1	3	2

**Table 8:** ANOVA analysis of the final performance scores.

Sr. No.	Source	Sum of Square	Degree of Freedom	F-Value	*p*-Value	Percentage Contribution
1	Guiding Hole Diameter	0.116	2	0.058	0.00225	52.85
2	Tool Rotation Speed	0.043	2	0.022	0.00083	19.52
3	Preheating Time	0.052	2	0.026	0.00100	23.45
4	Error	0.009	20			4.18

## Data Availability

Not applicable.
